# Measurements of Density of Liquid Oxides with an Aero-Acoustic Levitator

**DOI:** 10.3390/ma14040822

**Published:** 2021-02-09

**Authors:** Sergey V. Ushakov, Jonas Niessen, Dante G. Quirinale, Robert Prieler, Alexandra Navrotsky, Rainer Telle

**Affiliations:** 1 School of Molecular Sciences and Center for Materials of the Universe, Arizona State University, Tempe, AZ 85287, USA; anavrots@asu.edu; 2 Institut fuer Gesteinshuettenkunde/Mineral Engineering, RWTH Aachen University, 52062 Aachen, Germany; prieler@ghi.rwth-aachen.de (R.P.); telle@ghi.rwth-aachen.de (R.T.); 3 Neutron Scattering Division, Oak Ridge National Laboratory, Oak Ridge, TN 37830, USA

**Keywords:** levitation, rare earth oxides, zirconia, hafnia, melting, thermodynamics

## Abstract

Densities of liquid oxide melts with melting temperatures above 2000 °C are required to establish mixing models in the liquid state for thermodynamic modeling and advanced additive manufacturing and laser welding of ceramics. Accurate measurements of molten rare earth oxide density were recently reported from experiments with an electrostatic levitator on board the International Space Station. In this work, we present an approach to terrestrial measurements of density and thermal expansion of liquid oxides from high-speed videography using an aero-acoustic levitator with laser heating and machine vision algorithms. The following density values for liquid oxides at melting temperature were obtained: Y_2_O_3_ 4.6 ± 0.15; Yb_2_O_3_ 8.4 ± 0.2; Zr_0.9_Y_0.1_O_1.95_ 4.7 ± 0.2; Zr_0.95_Y_0.05_O_1.975_ 4.9 ± 0.2; HfO_2_ 8.2 ± 0.3 g/cm^3^. The accuracy of density and thermal expansion measurements can be improved by employing backlight illumination, spectropyrometry and a multi-emitter acoustic levitator.

## 1. Introduction

Most metal alloys are produced by melt processing, and thermodynamic and thermophysical properties of metallic melts have been systematically investigated for over a century. The developed ab initio and Calphad-based computational tools show spectacular results for the prediction of crystallization pathways and equilibrium phases for metal alloys [1]. Refractory oxide ceramics are usually produced by sintering, and application-driven incentives to study high temperature oxide melts have been largely limited to metallurgical slugs and glasses. The situation has changed with the application of additive manufacturing techniques to ceramic materials [2,3,4,5,6,7]. These techniques often involve laser melting, and their advance is hampered by a lack of data on oxide melts.

A plethora of techniques is available for high temperature study of metal alloys. The “exploding wire” technique [8,9] has been in development for more than 300 years and has also been adapted for electrically conductive carbides and nitrides [10,11,12,13]. In this method, pulse discharge through a metallic wire or conducting ceramic coating provides instantaneous heating and excludes any contamination from the container.

Electromagnetic [14] and electrostatic [15] levitation have been successfully used for contactless high temperature studies on metal alloys for decades [16,17,18,19,20,21,22]. Electromagnetic levitation was also applied to semiconductors after preheating; liquid silicon was studied extensively with this technique [20,23]. Image-based density measurements using levitation have been reported since the 1960s [24], and have been further refined using modern machine vision algorithms and applying Legendre polynomial fitting to the results of edge detection routines for volume calculation [23,25,26,27,28]. Modulated laser calorimetry on electromagnetically levitated melts was developed by Fukuyama et al. in 2007 [29]. Data on excess volume from image processing combined with data on excess heat capacities of mixing [30] provide a thermodynamic foundation for constructing realistic solution models for metallic alloys.

However, many refractory oxides are dielectrics and cannot be studied using exploding wire or electromagnetic levitation. Electrostatic levitation can be applied to dielectric materials, but it relies on surface charges and is challenging in terrestrial conditions. Most of the work on the application of electrostatic levitation to oxides was accomplished by the group at Tsukuba Space Center in Japan [31,32,33,34]. It culminated in the design of an electrostatic levitation furnace (ELF), which is currently in operation at the International Space Station [35]. The first results on the density of liquid Er_2_O_3_ and Gd_2_O_3_ were published in 2020 [36,37].

Aerodynamic levitation in a conical nozzle (CNL) [38,39,40,41] has been used extensively for oxide melts; however, it has limitations of limited sample visibility and large thermal gradient. The development of an aero-acoustic levitator (AAL) was funded by NSF and NASA and built by Intersonics Inc. in 1990 [42]. In this method, the sample is stabilized above the gas jet by acoustic forces that allow unimpeded access for multi-beam laser heating, pyrometer aiming and video recording.

Only two AAL instruments were commercially produced [43]: the first one with analog controls [44], which was operated in Japan [45,46,47]. The second one, used in this work, was built for RWTH Aachen University [48]. It enabled the first direct observation of liquid immiscibility between zirconia and silica-rich melts in the ZrO_2_-SiO_2_ system [49]. To the authors’ knowledge, this is the only instrument of its kind in operation to date. However, it will not be for long. Marzo et al. [50] made openly accessible a new acoustic levitator design using mass-produced acoustic transducers. This drastically reduces the cost of the development of new generation AAL. We anticipate that this innovation will result in a wider application of this technique to study oxide melts. In this work, we present measurements of the density of Y_2_O_3_, Yb_2_O_3_, YSZ, and HfO_2_ melts with an aero-acoustic levitator using machine vision algorithms developed for metal alloys.

## 2. Materials and Methods

### 2.1. Sample Synthesis

HfO_2_, Y_2_O_3_, and Yb_2_O_3_ oxide spheroids 2–3 mm in diameter were prepared by the melting of oxide powders obtained from Alfa Aesar (Ward Hill, MA, USA) with metals purity 99.98% or higher. The powders were sintered at 1500 °C in air for 5 h, then placed into a copper hearth and melted with a 400 W CO_2_ laser beam into irregularly shaped pieces surrounded by an unmelted powder bed. The resulting solid pieces were remelted in a conical nozzle aerodynamic levitator in Ar flow. Experiments were also performed on laser melted Y_0.05_Zr_0.95_O_1.975_ and Y_0.1_Zr_0.9_O_1.95_ samples prepared and characterized earlier for neutron diffraction experiments [51].

### 2.2. Measurement Procedure

**Figure 1:**
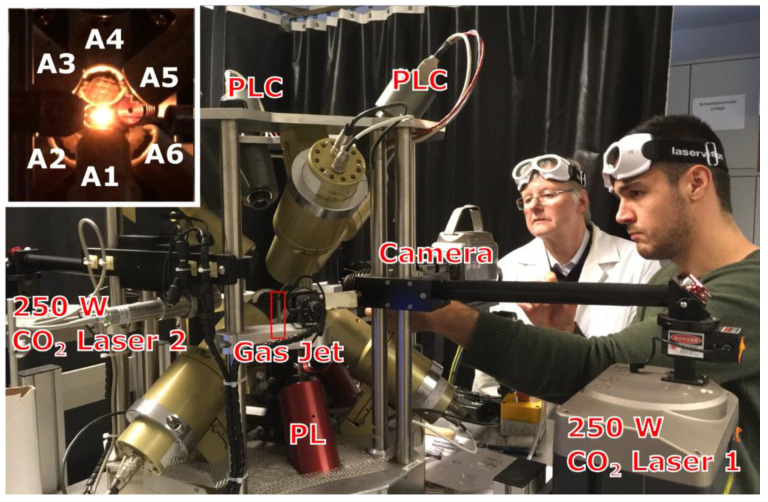
Operation of the aero-acoustic levitator at RWTH Aachen. J.N. is positioning the bead using an air pick above the gas jet for levitation. Inset: the sample bead heated with the dual laser beam, A1–A6 are frequency matched 22.2 kHz acoustic transducers controlled with input from positioning lasers and cameras (labeled PL and PLC, respectively).

The design of the aero-acoustic levitator used in this work (Figure 1) was described in detail by Nordine et al. [48]. The sample for levitation was positioned above the alumina tube heated to 550 °C, which serves as a gas jet. The levitation of the sample above the jet is stabilized by six acoustic transducers controlled with levitator software using a positioning system with three low power 808 nm solid state lasers. Experiments with Y_2_O_3_ were performed using N_2_ or Ar gas jets; due to high density of HfO_2_ and Yb_2_O_3_ samples their levitation was only possible using an Ar gas jet.

After levitation was established, the sample was heated to its melting temperature with two antiparallel 240 W CO_2_ laser beams (Synrad, WA, USA). The video was recorded using a Phantom V9.1 camera from Vision Resarch, Inc. (Wayne, NJ, USA) with an acquisition interval of 0.5–1 ms and exposure time of 20–200 µs. The temperature was recorded with a narrow band 650 nm Exactus pyrometer from BASF Corporation (Florham Park, NJ, USA). The pyrometer was operated with a 1-ms acquisition interval; the measurement spot size was set to 0.8 mm, and emissivity was set to 1. It was possible to record videos and cooling traces for the crystallization of Y_2_O_3_, Zr_0.95_Y_0.05_O_1.975_, and Zr_0.9_Y_0.1_O_1.95_ samples. This allowed correlation of the spike in density trace with recalescence peaks on crystallization, obtaining density values at melting temperature, and evaluating volume thermal expansion of the liquids. Levitated HfO_2_ and Yb_2_O_3_ melts became unstable after turning off the lasers and fell out of the field of view before recalescence peaks were captured with a pyrometer. This is attributed to their higher density. Levitation stability in AAL is discussed in detail by Nordine et al. [48].

### 2.3. Video Processing

Volume was calculated using a modification of the algorithm developed by Bradshaw et al. [25] as implemented by Bendert et al. [52]. As the videos were not filtered, the large temperature range and surface features of the molten samples made shape determination using edge detection routines difficult, so the video contrast was post-processed using open source software [53] to provide better definition. The numerical routines were used to calculate volume assumed symmetry about the vertical axis. This is not necessarily the case with aero-acoustically levitated droplets, which may be slightly asymmetric and experience a slight precession, introducing some additional uncertainty into the measurement. The implementation of multi-emitter acoustic levitator design [50] may reduce this uncertainty.

Camera calibration was performed by imaging commercially obtained machined Al_2_O_3_ spheres 3.27 mm in diameter levitated without laser heating. The video was processed using the same procedure as for the laser-heated molten oxides. The variation in calculated volume from the machine vision algorithm did not exceed 1%. The main contribution to calibration uncertainty comes from measurements of diameter and sphericity of the Al_2_O_3_ sphere (taken as ±0.025 mm). The total uncertainty in volume from camera calibration was estimated as ±3%, from ±2.3% uncertainty in volume of the calibration standard and ±0.3% variation in volume from video edge detection procedure.

Correlation of the pyrometer trace with the video recording and density curve obtained from video analysis was performed manually. The moment of turning off the lasers is clearly observed from the disappearance of the bright spot on the molten sample. The onset of crystallization was evident from the video from a sudden increase in sample brightness—sample “flash” or recalescence, caused by reheating the sample on crystallization by released heat of fusion.

## 3. Results and Discussion

**Figure 2:**
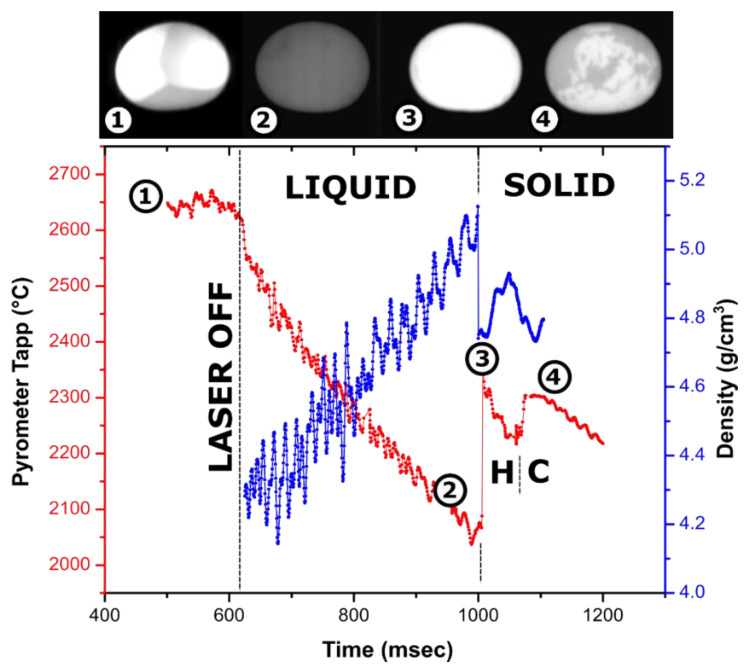
Cooling trace of a 59-mg Y_2_O_3_ sample (~2.5 mm in diameter) melted in an Ar jet in an aero-acoustic levitator with overlayed density measurements and video frames (lateral view): (1)—molten droplet heated laterally with two laser beams which are visible as bright spots; (2)—undercooled liquid before crystallization; (3)—recalescence or “flash” on crystallization; (4)—phase transformation from high temperature hexagonal to cubic bixbyite phase. The brightness of images (2–4) was adjusted by the same degree for visibility. A video fragment is provided in Supplementary Materials (Video S1).

**Figure 3:**
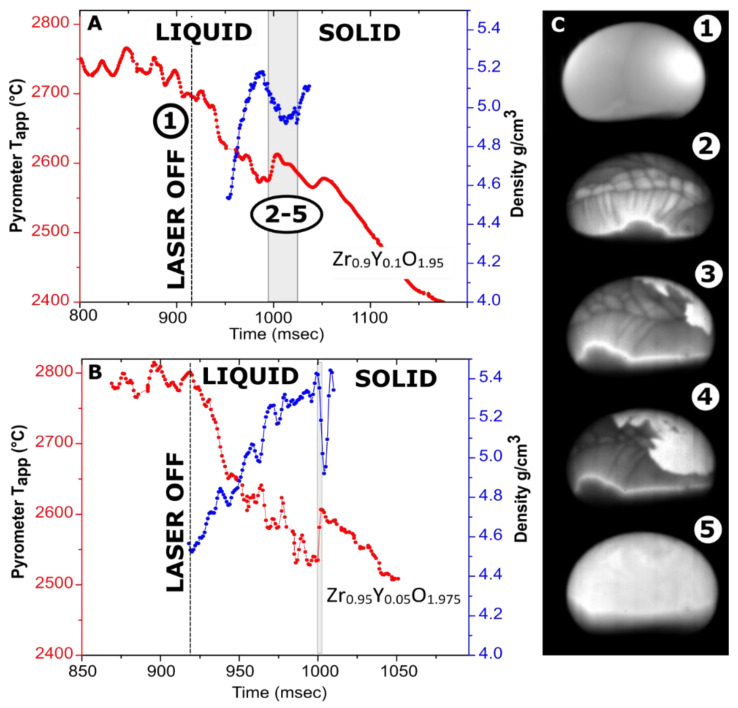
Cooling traces with overlayed density measurements. (**A**) 119.5-mg Zr_0.9_Y_0.1_O_1.95_ sample levitated in Ar; (**B**) a 32-mg Zr_0.95_Y_0.05_O_1.975_ sample levitated in nitrogen; (**C**) video frames for the Zr_0.9_Y_0.1_O_1.95_ sample (~3.5 mm in diameter): (1)—before turning off the lasers (visible as bright spot); (2–5)—113, 132, 135 and 151 ms after turning off the laser. The brightness was adjusted individually for every frame. A video fragment is provided in Supplementary Materials (Video S2).

In experiments performed on Y_2_O_3_, Zr_0.95_Y_0.05_O_1.975_, and Zr_0.9_Y_0.1_O_1.95_, it was possible to record recalescence on cooling traces and videos. This allowed accurate temperature correlation of density values and evaluation of thermal expansion of the liquid. Stable levitation of HfO_2_ and Yb_2_O_3_ through recalescence was challenging, and we did not succeed in recording videos of recalescence. However, considering 3% uncertainty from calibration, we attribute the measured values for HfO_2_ and Yb_2_O_3_ to the melting temperatures. Matched temperature–density profiles are shown in Figure 2 and Figure 3. The obtained density and thermal expansion data are summarized in Table 1, together with relevant reference values.

### 3.1. Measurements on Y_2_O_3_

The density profile overlayed with the cooling trace of the Y_2_O_3_ droplet is shown in Figure 2. The ~300 °C temperature rise on recalescence gives the magnitude of the observed undercooling of liquid Y_2_O_3_. Recalescence can be pinpointed as a flash on the video, and the density profile shows a sharp decrease due to reheating of the sample. After crystallization onset, the density calculations from the bead dimension are not meaningful since the surface of the droplets crystallizes first, and cavities are formed on further crystallization of core parts of the sample.

The first phase to crystallize from Y_2_O_3_ melt is known to be a hexagonal phase, common to lanthanide oxides [54,55,56,57]. It is stable in a narrow ~100 °C temperature range and undergoes a transition to cubic bixbyite, which is stable at room temperature. This transition is clearly seen on the cooling trace as a second peak with a smaller ~100 °C rise corresponding to undercooling on hexagonal (H-type) to cubic phase transformation and a plateau at a temperature ~100 °C lower than the recalescence peak. Apart from the “flash” of the bead due to temperature increase, crystallization of the H phase is not clearly distinguished in videos, indicating its emissivity is similar to that of the liquid.

On turning off the laser, the density of liquid Y_2_O_3_ increases from 4.3 g/cm^3^ at 2650 °C to 5.1 g/cm^3^ at 2100 °C. The density of the liquid at the melting temperature is estimated at 4.6 ± 0.15 g/cm^3^. For the experiment shown in Figure 2, observed fluctuation in refined density is likely due to the non-symmetrical oscillation of the molten sample in the acoustic field, which ceases after crystallization. In addition to the experiment shown in Figure 2, density was refined from experiments on three more Y_2_O_3_ samples, 56–80 mg in weight, using Ar and N_2_ for levitation. The refined values varied from 4.3 to 4.7 g/cm^3^. In several experiments, the scatter in volume from video processing was as low as ±0.01 g/cm^3^. We were not able to correlate other measurements with the temperature trace on cooling; however, the observed variation is consistent with the density value from Figure 2. Volume thermal expansion of liquid Y_2_O_3_ at melting temperature was estimated as (3 ± 1) × 10^−4^ K^−1^.

### 3.2. Measurements on Zr_0.9_Y_0.1_O_1.95_ and Zr_0.95_Y_0.05_O_1.975_

The density profiles overlayed with cooling traces from Y-doped zirconia samples are shown in Figure 3. Undercooling on the crystallization of Zr_0.9_Y_0.1_O_1.95_ and Zr_0.95_Y_0.05_O_1.975_ does not exceed 50 °C and 80 °C, respectively. The recalescence step is much more pronounced for a smaller sample. In previous experiments on these compositions in an aerodynamic levitator, no recalescence peaks were detected [51], likely due to the larger sample gradient.

The video stills from the crystallization of Zr_0.9_Y_0.1_O_1.95_ (Figure 3C) have interesting features. The Marangoni flows on the cooling of Zr_0.9_Y_0.1_O_1.95_ are more pronounced than for Y_2_O_3_ but are not observed at the bottom of the sample. The Marangoni flows are caused by temperature or composition-related gradients in surface tension. Calorimetry experiments indicated the possibility of oxygen dissolution in liquid ZrO_2_ and HfO_2_ [63]. In our experiments, the melting of Zr_0.9_Y_0.1_O_1.95_ was accomplished in an air–argon mixture, with argon flow provided by an auxiliary gas jet. The lower oxygen fugacity at the bottom of the sample impinged upon by the argon jet could be a plausible reason for this behavior.

In the case of congruent crystallization, one would expect that a solid phase would first appear at the bottom surface of the sample due to additional cooling by the argon jet. This is the case for the C–H transformation in Y_2_O_3_ (Figure 2). However, this is not what happens in Zr_0.9_Y_0.1_O_1.95_, in which the bottom of the bead seems to crystallize last. This supports the hypothesis of variable oxygen content in the melt. The densities of Zr_0.95_Y_0.05_O_1.975_ and Zr_0.9_Y_0.1_O_1.95_ at melting temperatures were estimated at 4.9 and 4.7 g/cm^3^, respectively. The difference between compositions is within the assigned experimental uncertainty of ±0.2 g/cm^3^.

### 3.3. Comparison with Previously Reported Density Values

In Table 1 the density and thermal expansion coefficient values for liquid oxides measured in this work are listed together with four types of previously published density data: (i) measurements in aerodynamic levitator by Granier and Heurnault’s [59], Kohara et al. [65], and Kondo et al. [66]; (ii) measurements with the electrostatic levitator on board of International Space Station [36,37]; (iii) refinement from pair distribution function analysis (PDF) of synchrotron X-ray scattering [62]; (iv) ab initio molecular dynamic computations [60,63].

Granier and Heurnault reported the density of liquid alumina, yttria, and several lanthanide sesquioxides [59,69]. They performed the measurements on photographs of laser-heated droplets levitated in a conical nozzle aerodynamic levitator (CNL). Their value for Al_2_O_3_ density at melting temperature is about 10% lower than most of the previous measurements [69]. Granier’s values for Y_2_O_3_ and for Yb_2_O_3_ are 4–5% lower than those measured in this work.

Density measurements for Gd_2_O_3_ and Er_2_O_3_ were recently performed by a Japanese group with an electrostatic levitator furnace (ELF) at the International Space Station (ISS) [35,36,37]. The electrostatic levitation in microgravity conditions ensures the absence of disturbances by acoustic waves or by gas flow, resulting in a perfectly spherical shape of the levitating droplet. Notably, Granier’s values for Gd_2_O_3_ and Er_2_O_3_ from CNL are also 4–7% lower than measured in ELF at ISS (Table 1).

There is a simple explanation for this discrepancy if one considers how measurements were performed in Granier’s and Heurnault’s study [59]. In the early version of the aerodynamic levitator they used, the sample was completely surrounded by the nozzle. The photographs were taken from the top, and density values were calculated assuming spherical shape of the sample. However, the shape of the levitated droplet is not an ideal sphere but an oblate spheroid. This can be clearly seen from the lateral view of molten samples in an aero-acoustic levitator (Figure 2). Using top view and assumption of spherical shape would overestimate the volume and underestimate the density. In terrestrial measurements, the degree of oblateness of the spheroid depends on the surface tension of the melt. Melting temperatures of Y_2_O_3_, Yb_2_O_3_, Gd_2_O_3_, and Er_2_O_3_ are ~400 °C higher than Al_2_O_3,_ and they are expected to have higher surface tension. For these oxides, the bead of the same dimension will be closer to spherical shape than in the case of Al_2_O_3_. This is consistent with better agreement of measurements for these oxides with Granier’s values.

Kohara et al. [65] also used CNL for measurements of density for liquid ZrO_2_, however they employed a very shallow nozzle which allowed the side view of the sample. Their value for density and thermal expansion of ZrO_2_ (5.05 g/cm^3^ and 1.8 × 10^−4^ K^−1^) is the same within uncertainty as our results for Zr_0.95_Y_0.05_O_1.975_ (4.9 ± 0.2 g/cm^3^ and (2 ± 1) × 10^−4^ K^−1^). The value measured for liquid HfO_2_ density in this work (8.2 ± 0.3 g/cm^3^) is in excellent agreement with the density reported by Gallington et al. [62] (8.16 g/cm^3^) from refinement of total synchrotron X-ray scattering on liquid HfO_2_.

Liquid ZrO_2_ density from ab initio molecular dynamic (AI MD) calculations [63] coincides with our value; however, the Y_2_O_3_ value from computations is 10% lower than measured, and for HfO_2_ and Yb_2_O_3_, it is 4–6% higher. The comparison of absolute density values from computations with experiment is compromised by uncertainty in absolute temperature in AI MD simulations and underlying assumptions such as choices of exchange-correlation functionals and size of the simulation system.

### 3.4. Temperature Measurements

In this work, we correlated high-speed video recording with recalescence peak to provide density and thermal expansion values in the proximity of melting temperature. This approach assumes that peak temperature on recalescence is close to the melting point and does not require knowledge of absolute values of the sample temperature. Thus, our measurements did not require knowledge of emissivity, but relied on known melting temperatures of measured oxides. The only assumption made about emissivity is that it does not change substantially for measured liquid oxides around the melting temperature. However, temperature calibration is a paramount issue for measurement of thermal expansion above the melting temperature and for determination of unknown temperatures of melting and phase transformations. Below we discuss methods of estimation of emissivity values and effective emissivities calculated for measured samples.

Temperature measurements with a single-color pyrometer require knowledge of spectral emissivity and its temperature dependence. These data for refractory oxides above 2000 °C are fragmentary [70]. For opaque materials, the sum of emissivity and reflectivity at given wavelength must be equal to one. From careful measurements of reflectivities at 650 nm in a solar furnace, Yamada and Noguchi [71] obtained emissivities for Y_2_O_3_ and ZrO_2_ at melting temperatures as 0.92 ± 0.005, and 0.89 ± 0.005. In an earlier study [72], the same group reported 650 nm emissivity values for HfO_2_ and Al_2_O_3_ at freezing points as 0.91 and 0.96, but without estimation of the uncertainties.

Nordine et al. [48] suggested that reflectivity of opaque melts can be estimated from the refraction index of the solid and emissivity can be calculated using the following relationship:

(1) $$\epsilon \left(\lambda \right)=1-r\left(\lambda \right)=1-\frac{{\left(n-1\right)}^{2}}{{\left(n+1\right)}^{2}}$$

where *r* is the reflectivity and *n* is the refractive index at the given wavelength λ This approach neglects temperature dependence of emissivity and changes in emissivities between solid and liquid phases. However, in the case of Al_2_O_3_ for which high temperature data are available [73] these changes are small.

The videos indicate that the melt is opaque at visible wavelengths, and therefore the transmittance can be neglected. Emissivity for different materials is calculated in Table 2 based on Equation (1). The differences from the available values reported by Noguchi [71] from direct measurements for liquid oxides do not exceed 3%. Thus, this approach can be used for future measurements.

When the melting temperatures are known or independently measured, the common approach is to estimate effective emissivity *ε_λ_* at melting temperature using Wien’s approximation:

(2) $$\frac{1}{T}=\frac{1}{T_{A}}+\frac{\lambda }{C_{2}}ln\left(\varepsilon_{\lambda }\right)$$

where *T* is the melting temperature, *T_A_* is the apparent melting temperature (measured by pyrometer), and *C*_2_ is Planck’s second radiation constant. The temperature dependence of emissivity is usually neglected, and the obtained value for effective emissivity can be used to correct the apparent temperature of the melt in the range of the measurements.

In dynamic measurements of phase transformations, metastability is common on cooling but not on heating. Thermal arrests on melting cannot be unambiguously distinguished on heating with a continuous wave (CW) laser. On cooling of oxide melts, crystallization onset is usually below equilibrium melting temperature. This undercooling is more pronounced in levitated samples in the absence of any solid–liquid interfaces. The crystallization of undercooled melt results in a peak in temperature-recalescence which can be visually observed as a “flash”. If on recalescence the sample is reheated to the equilibrium melting temperature, the true thermal arrest can be observed. Ideally, its temperature should be used for emissivity calculation at the melting point.

In our experiments, we do not observe thermal arrest on recalescence. It is not surprising for 2–3 mm beads with melting temperatures above 2400 °C. Due to the relatively large surface to volume ratio and high temperatures, heat transfer by radiation does not allow sample reheating to melting temperature by released heat of fusion.

Table 3 lists emissivity values calculated from Equation (2), taking the maximum temperature of recalescence peak as the apparent melting temperature. The values calculated based on this method will always underestimate emissivity when the melting point is not reached; therefore, they should be seen as a lower boundary. Measurements on a curved surface will also underestimate emissivity; thus, these differences are expected. While single-color pyrometry remains the fastest and most sensitive technique, it must be noted that approaches which do not require knowledge of emissivity for temperature measurements are well established, such as direct measurements of reflectivity [78] and spectropyrometry [79,80,81].

## 4. Conclusions and Future Directions

This work demonstrates that reasonable values for density of liquid oxides can be obtained from high-speed videography measurements with an aero-acoustic levitator. Volume change on melting cannot be directly obtained from these experiments. While videography can be used on solids, samples in this work were prepared by laser melting and contained cavities formed on solidification. However, volume change on melting can be derived by combining density data for liquids with thermal expansion data on solids from X-ray diffraction [51,57,82].

The accuracy of the measurements can be significantly improved by back illumination of the levitated samples, combined with appropriate filters on the camera [27,34], which is known to aid edge detection in image processing. The multi-emitter single-axis acoustic levitator introduced by Marzo et al. [50] allows levitation of non-spherical samples with density up to 6.5 g/cm^3^. The adaptation of a new multi-emitter design for laser heating and density measurements can simplify levitation, decrease the deformation of the liquid sample by acoustic waves, and eliminate or drastically reduce the need for auxiliary aerodynamic support of the sample.

The measurements of change in density with melt composition can be used to obtain the excess volume of mixing in the liquid state for multicomponent systems and derive realistic thermodynamic mixing models, as demonstrated by Fukuyama et al. [30] for metal alloy systems. Such measurements for key refractory oxide systems will be the subject of future studies.

## Figures and Tables

**Table 1:** Density and volumetric thermal expansion coefficient (TEC) for liquid oxides at melting temperatures (*T*_m_) measured in this work compared with previously published data.

Composition	*T*_m_, °C [Ref.]	Density g/cm^3^	TEC 10^−4^ K^−1^	Method ^†^	Ref.
Y_2_O_3_	2431 [58]	4.6 ± 0.15	3 ± 1	AAL	This work
		4.42	1.9	CNL↓	Granier 1988 [59]
		4.15 ^‡^	4.5	AI MD	Kapush 2017 [60]
HfO_2_	2800 [61]	8.2 ± 0.3	-	AAL	This work
		8.16		PDF	Gallington 2017 [62]
		8.7 *		AI MD	Hong 2018 [63]
Zr_0.95_Y_0.05_O_1.975_	2730 [64]	4.9 ± 0.2	2 ± 1	AAL	This work
Zr_0.9_Y_0.1_O_1.95_	2750 [64]	4.7 ± 0.2	3 ± 1	AAL	This work
ZrO_2_	2710 [61]	4.9	0.9	AI MD	Hong 2018 [63]
		5.05	1.8	CNL→	Kohara 2014 [65]
		4.69 ± 0.23	0.9	CNL→	Kondo 2019 [66]
Yb_2_O_3_	2434 [67]	8.4 ± 0.2	-	AAL	This work
		7.94	0.9	CNL↓	Granier 1988 [59]
		8.75	4.5	AI MD	Fyhrie 2019 [68]
Er_2_O_3_	2417 [67]	8.17 ± 0.16	1.0	ESL(ISS)	Koyama 2020 [37]
		7.60	0.4	CNL↓	Granier 1988 [59]
Gd_2_O_3_	2420 [67]	7.24 ± 0.14	0.7 ± 0.2	ESL(ISS)	Ishikawa 2020 [36]
		6.93	1.5	CNL↓	Granier 1988 [59]

^†^ Methods abbreviation: AAL—aero-acoustic levitation; CNL ↓—conical nozzle aerodynamic levitation top view; CNL →—idem., side view; PDF—refined from experimental pair distribution function; ESL(ISS)—electrostatic levitation at international space station; AI MD—ab initio molecular dynamic computations. ^‡^ The density value from calculations at 2377 °C. * The density value from calculations at 2827 °C.

**Table 2:** Calculated emissivity based on the reported values for the refractive index (*n*).

Composition	*T*, °C	*n*	*λ*, nm	*ε*_calc_	Reference for (*n*) Value
Al_2_O_3_	25	1.78	632.8	0.92	Krishnan 1991 [73]
Y_2_O_3_	25	1.92	650	0.90	Nigara 1968 [74]
HfO_2_	25	2.08	600	0.88	Hu 2003 [75]
Yb_2_O_3_	25	1.94	643.8	0.90	Medenbach 2001 [76]
ZrO_2_ 12 mol% Y_2_O_3_	25	2.15	650	0.87	Wood 1982 [77]

**Table 3:** Emissivity values calculated from assumption that the recalescence peak reaches the melting temperature (*T*_m_).

Composition	*T*_m_ °C [Ref.]	*ε*_calc_ (at *T*_m_ 650 nm)	Ref.
Y_2_O_3_	2431 [58]	0.8	This work
Zr_0.95_Y_0.05_O_1.975_	2730 [64]	0.68	This work
Zr_0.9_Y_0.1_O_1.95_	2750 [64]	0.65	This work

## Data Availability

The data presented in this study are available on request from the corresponding authors.

## Supplementary Materials

The following are available online at https://www.mdpi.com/1996-1944/14/4/822/s1, Video S1: Crystallization of 58.79 mg Y_2_O_3_ sample (500 µs acquisition interval, 200 µs exposure, exported to AVI format at 50 frames per second), Video S2: Crystallization of 119.50 mg Y_2_O_3_ sample (1000 µs acquisition interval, 40 µs exposure, exported to AVI at 50 frames per second)
